# Health system interventions and responses to anti-microbial resistance: A scoping review of evidence from 15 African countries

**DOI:** 10.1371/journal.pgph.0003688

**Published:** 2024-09-18

**Authors:** Jacob Albin Korem Alhassan, Clement Kamil Abdallah

**Affiliations:** 1 Department of Community Health and Epidemiology, College of Medicine, University of Saskatchewan, Saskatoon, Canada; 2 Ad Astra Foundation, Tamale, Ghana; Amrita Institute of Medical Sciences, INDIA

## Abstract

The global rise in antimicrobial resistance (AMR) is claiming the lives of more than 1.2 million people each year. According to the World Health Organization (WHO) this global health crisis is particularly acute in Africa, largely due to fragile and underfunded health systems. Efforts to combat this public health threat have led to the implementation of health system interventions worldwide aimed at managing and containing the spread of AMR. However, the literature on the real time impacts and the barriers that hinder the implementation of these interventions in the African context is limited. The objective of this scoping review was to identify AMR interventions in African health systems, their impact, and the challenges of the implementation. Drawing on Muka and colleague’s 24 step approach for scoping reviews, two major public health databases (PubMed and Global Health) were searched for articles in accordance with the PRISMA guidelines resulting in 4,783 records. Screening and retrieval of articles was done using Rayyan software based on specified inclusion criteria and 36 articles included in the final list. These articles were synthesized after extracting specific data on AMR interventions and their impact on African health systems. The review identified four broad impacts of AMR interventions including 1. Reduction in antibiotics use, 2. Increased adherence to guidelines and protocols, 3. Enhanced laboratory-based AMR surveillance, 4. Development of antimicrobial stewardship (AMS) Action Plans and Teams. However, challenges such as poor laboratory infrastructure, logistical challenges, poor financial commitment and inadequate education and training were identified as challenges impeding the successful implementation of AMR interventions in Africa. Our findings reveal a range of successful AMR interventions in African health systems although infrastructural and financial challenges remain. Better standardization and reporting of AMR diagnosis while leveraging the available information is needed to improve the optimization of treatment guidelines across Africa.

## 1. Introduction

Antimicrobial Resistance (AMR) has become a global public health crisis, responsible for over 1.2 million deaths annually [1]. The World Health Organization (WHO) has described the spread of AMR as unparalleled, affecting both High Income Countries (HICs) and low-and-middle-income countries (LMICs) [2]. AMR has posed a significant challenge to science and medicine ever since the discovery of antibiotics. It is influenced by a variety of biological and social factors, but is driven primarily by misuse and overuse by patients as well as inappropriate prescription of antibiotics by healthcare providers [3]. The adverse effects of AMR are particularly pronounced in Africa, where under-resourced healthcare systems and a high disease burden exacerbate the problem.

Many countries have taken steps to safeguard the effectiveness of last-line antibiotics by implementing AMR interventions. For instance, China has banned the use of last-line antibiotics in animal feed [4]. However, a team of researchers from Oxford University recently found that reducing the use of antibiotics in animal farms alone is insufficient to curb the rapid spread of AMR [5]. Moyo et al [6] have suggested that achieving greater success in combating AMR depends mainly on the success of health system level interventions.

The World Health Organisation (WHO) has recommended six crucial interventions necessary at the health system level to combat AMR especially in resource limited settings including antimicrobial leadership; training and education for health service providers and patients; antimicrobial guidelines and protocols; feedback on antibiotics use; reporting; and accountability and responsibility [7]. Successful AMS programmes require multisectoral and multifaceted approaches involving patients, Health Care Professionals (HCPs) and financial commitment from governments.

The Africa Centres for Diseases Control and Prevention (Africa CDC) has determined that the many health system interventions related to AMR in Africa are currently deficient and incapable of effectively combating AMR. Extant research indicates that the majority of laboratories across Africa are ill-prepared for AMR testing, resulting in suboptimal clinical decisions regarding antibiotic prescriptions [8]. Notwithstanding the foregoing, country-specific health system interventions in Africa reveal some progress. For example 93% of the 4,841 laboratories in Ghana have successfully conducted AMR blood culture surveillance to support antibiotics prescription decisions [9].

The existing body of literature on AMR interventions in Africa have focused on AMR policies, national guidelines and action plans by governments [3,10–15] and civil society organizations [16]. Most of the existing reviews from our preliminary literature search, described the causes and burden of AMR in Africa [17], or restricted themselves to regions within Africa like West Africa [18] or particular countries like Ghana [19]. While African countries have implemented health system interventions to address AMR [6], there are limited reports documenting their real-time impacts and implementation challenges. The primary aim of this scoping review was to describe health system level AMR interventions in Africa, their impacts, and the difficulties encountered during implementation. Our intention is to provide policymakers with an up-to-date assessment of the state of AMR interventions in Africa. The review was structured around two primary research questions: 1. What are the impacts of AMR health system interventions in Africa? 2. What are the challenges encountered during implementation of AMR health system interventions in Africa?

## 2. Methods

Our review was guided by the 24-step approach suggested by Muka et al. [20]. This approach was selected because it offers a comprehensive guide to evidence synthesis to ensure that reviews are informative and reliable. We adapted the 24 steps as appropriate and specifically included the following: 1. Define research question; 3. Define the search strategy; 4. Define selection criteria; 5. Design data collection form; 7. Run the search strategy in multiple databases; 9. Eliminate duplicates; 10. Two reviewers to screen titles and abstracts; 11. Collect, compare and select studies for retrieval; 12. Retrieve full text and apply selection criteria; 15. Compile final selected full texts and draw flow chart; 18. Conduct descriptive synthesis; and 24. Update and report. [20].

**Fig 1:**
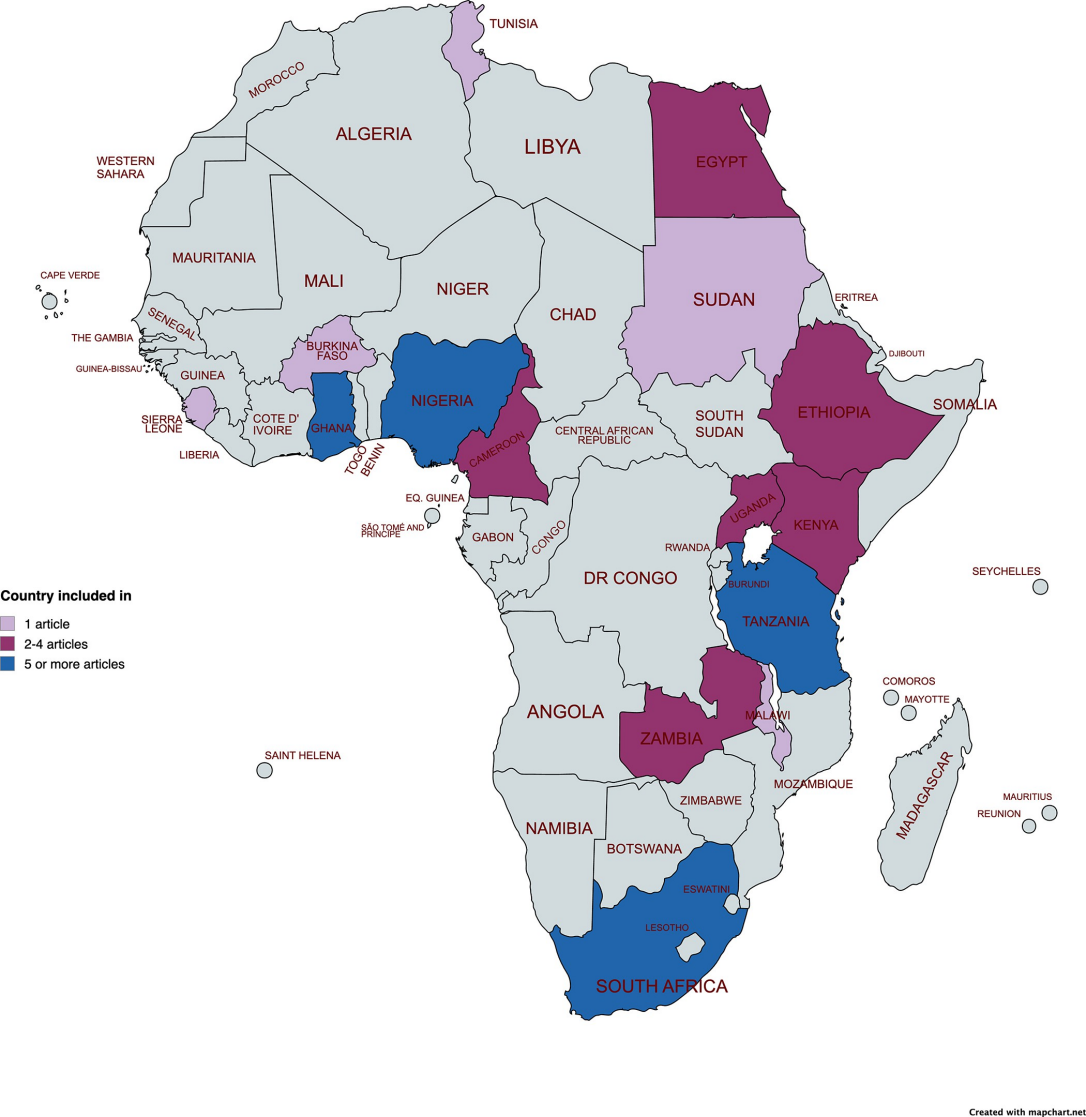
Geographical distribution of included studies. The source of the base map shapefile available at mapchart.net/africa.html and published with permission under creative commons (see mapchart.net/terms.html).

The purpose of the review was to examine health-system AMR interventions implemented across Africa and the achievements, and challenges encountered in the implementation of these interventions. Only health-system based interventions implemented in Africa are covered for this review (Fig 1); interventions in other parts of the world were excluded.

On July 27, 2023, two databases (Global Health and PubMed) were searched in consultation with an academic librarian. The following search terms were used to retrieve articles from the databases: (Drug Resistance OR Multiple, Bacterial/ OR Drug Resistance, OR Microbial/ OR Antimicrobial Resistance*) AND (health system* OR health equity or "human health" OR "communication" education or training OR "capacity building" OR orientation OR campaign OR surveillance OR sanitation OR hygiene OR "infection prevention control" OR IPC OR "antibiotic stewardship" OR "antimicrobial stewardship" OR "one health" AND “African Countries”).

The selection criteria for studies included: 1. Publication in English, 2. Empirical full-text peer-reviewed articles, 3. Studies focused on AMR interventions in African health systems, 4. Studies examining the impact and challenges of AMR interventions, and 5. Published after 2012.

**Fig 2:**
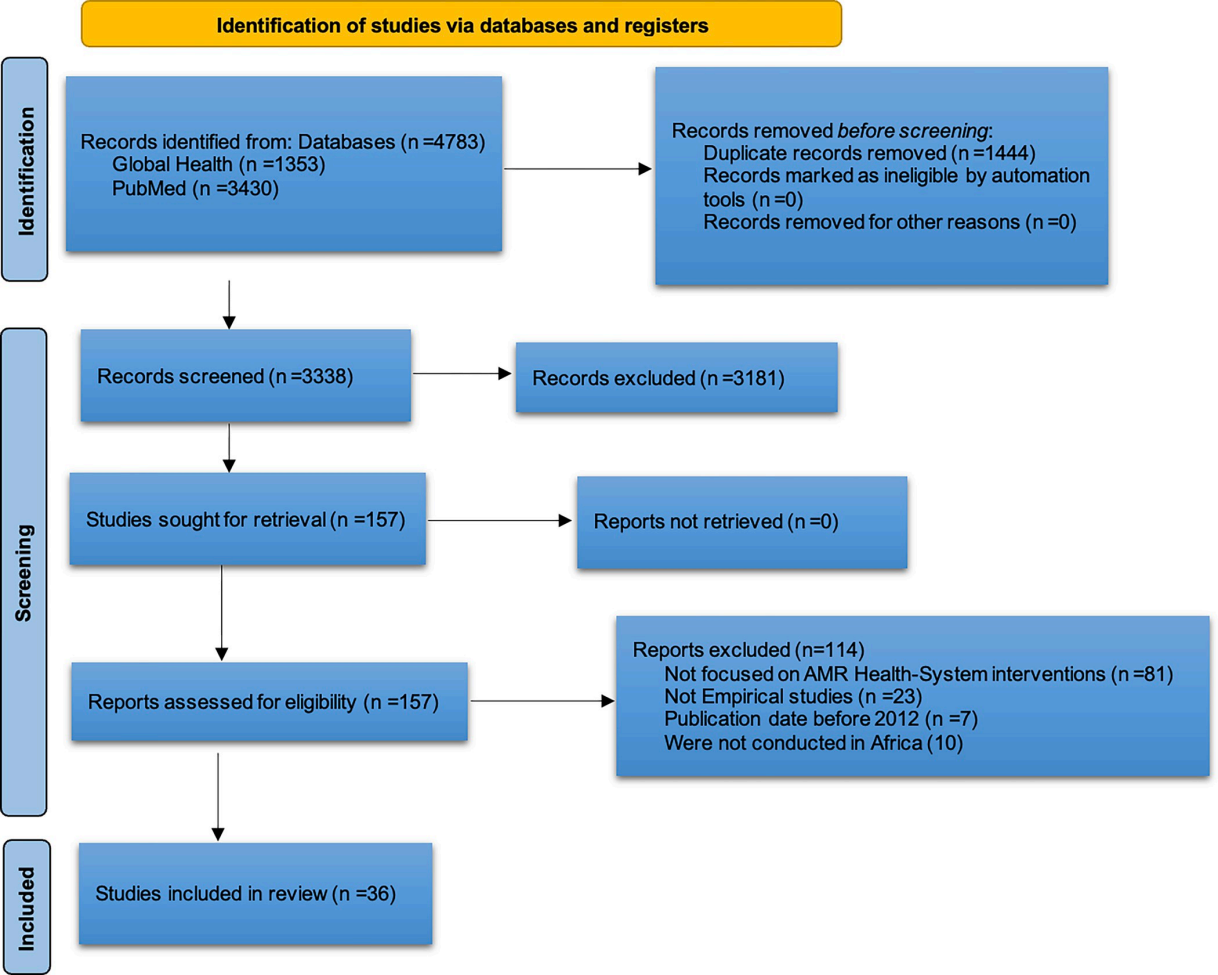
PRISMA diagram describing literature search.

The database search results were exported to Rayyan software for further review. Twelve steps were used consistent with the 24-step approach to review articles. The review was conducted by two reviewers and included articles are reported using the Preferred Reporting Items for Systematic Reviews and Meta-Analyses (PRISMA) diagram below (Fig 2). Once data charting was completed, we synthesized results by identifying the most commonly described health system interventions their impacts, and the challenges of implementation. We then categorized these based on similarity. For example, among included studies, we grouped together interventions where the focal point was education of healthcare workers, brought together the studies that emphasized infection prevention and control and grouped together those studies that emphasized action plans etc. We then synthesized these studies focused on how similarly they described health system interventions across these domains to provide a summary of the most common interventions. We used a similar approach to identify challenges and impacts of interventions related to AMR control in Africa.

## 3. Findings

The search yielded a total of 4,783 articles from Global Health (1,353) and PubMed (3,430). After removing 1,444 duplicates, 3,338 articles were screened based on their titles and abstracts. This process resulted in 157 articles being shortlisted for a full text review to determine whether they met the inclusion criteria. Ultimately, 36 peer-reviewed articles were included in the final review (see Table 1 and Fig 2), while 121 articles did not meet the inclusion criteria because they did not focus on healthcare-system AMR interventions (81); they were systematic reviews (23); their publication date was before 2012 (7) or they were not conducted in Africa (10). The included studies were conducted in 15 African countries (see Fig 1), specifically in Western [21–27], Middle [28,29], Eastern [29–38], Southern [39–45] and Northern Africa [46–49]. The included studies used diverse research methodologies, including qualitative, quantitative, and mixed methods, often involving longitudinal or experimental designs.

**Table 1:** Summary of included studies.

Author(s) and Year	Country	Methodology			Identified AMR Interventions					Impact of Intervention				Challenges of Implementation	
		Qual	Quant	Mixed	Education, Training and Capacity Building	Infection Prevention and Control	Monitoring and Evaluation	AMS Team and Support Facilities	Delay/Backup Prescription	Reduction in the use Antibiotics	Adherence to Guidelines and Protocols	Enhance Lab-based Surveillance	Development of AMS Action Plan and Team	Lack of Resources	Inadequate Training and Education
Sibande et al, 2022 [39]	Malawi		X				x				x				x
Boyles et al. 2017 [45]	South Africa		X		X		X			X		X			
Nayiga et al, 2022 [33]	Uganda	X					X							X	
Egwuenu et al, 2022 [21]	Nigeria			X				X						X	
Chaplain et al, 2022 [34]	Uganda	X					X	X				X		X	X
Nkosi and Sibanda, 2021 [40]	South Africa		X			X	X				X				
Chukwu et al, 2021 [22]	Nigeria			X			X				X		X	X	
El-Sokkary et al, 2020 [47]	Egypt	X					X			X		X		X	
Sangeda et al, 2020 [32]	Tanzania		X				X			X				X	
Bassiouny et al, 2019 [46]	Egypt			X			X				X				
Hall et al. 2020 [35]	Tanzania	X						X					X	X	
Aika and Enato, 2022 [57]	Nigeria	X						X					X		X
Sneddon et al. 2020 [52]	Ghana		X		X					X	X				
Abrudan et al. 2021 [42]	Nigeria, South Africa and Sudan		X		X							X			
Lakoh et al. 2023 [53]	Sierra Leone		X		X			X					X	X	
Ola-Bello et al. 2023 [55]	Nigeria	X								X	X				X
Darkwah et al. 2021 [25]	Ghana		X				X			X					
D’Arcy et al. 2021 [36]	Ghana, Uganda, Zambia and Tanzania		X		X								X	X	
Junaid et al. 2018 [50]	South Africa	X			X								X		
Amponsah et al 2023 [24]	Ghana		X		X	X	X			X		X			
Ibrahim et al. 2018 [51]	Ethiopia	X			X									X	
Bellazreg et al. 2022 [48]	Tunisia		X					X		X					
Ghebrehewet et al 2020 [26]	Ghana		X						X	X					
Valimba et al. 2014 [37]	Tanzania		X		X					X				X	
Peters et al 2021 [43]	South Africa		X				X			X				X	
Hadley and Beard, 2019 [30]	Zanzibar, Tanzania		X							X					X
Fadare et al, 2019 [56]	Nigeria		X				X			X			X		
Chetty et al, 2022 [44]	South Africa		X				X	X			X				
Yopa et al. 2023 [28]	Cameroon			X			X						X		X
Engler et al. 2020 [41]	South Africa		X		X		X							X	
Kerr et al. 2021 [38]	Ghana, Tanzania, Zambia and Uganda		X		X		X			X	X			X	
Bessat, Zonon and D’Acremont, 2019 [23]	Burkina Faso	X						X		X					
McKnight et al, 2019 [31]	Kenya	X					X	X		X				X	
Trollip et al. 2022 [29]	Cameroon, Ethiopia and Kenya		X					X							X
Oshun et al. 2021 [27]	Nigeria			X	X					X				X	
Shawki, et al 2021 [49]	Egypt		X			X		X		X					

Health-system interventions to combat AMR include a wide range of activities aimed at reducing AMR in communities and hospitals. After a comprehensive review of the selected articles, AMR interventions in healthcare systems were synthesized and classified into four broad types including: 1. Training and Capacity Building, 2. Infection Prevention and Control, 3. Monitoring and Evaluation (Guidelines, audit and Feedback), and 4. AMS Team and Support Facilities. In addition, three common challenges were identified that hinder the implementation of the interventions including: 1. Lack of resources; 2. Inadequate training and education and 3. Other challenge4s. Further, the implementation of most interventions was impactful and yielded positive results in the control of AMR. However, few interventions counterintuitively resulted in the abuse of antibiotics [33].

### 3.1 AMR health-system interventions

Health system AMR interventions are part of strategies and measures implemented by governments to address the growing problem of AMR. Five main types of interventions were identified ranging from interventions aimed at improving AMR surveillance facilities to those aimed at building human resource capacity to ensure monitoring of antibiotic consumption.

#### 3.1.1 Education, training and capacity building

One of the most common interventions in the included studies is education and training. Education provides HCPs, patients and the public with valuable information about AMR [50], the importance of infection prevention and the appropriate use of antibiotics. Training also provides physicians and pharmacists with timely information on updated global guidelines and Antimicrobial Surveillance (AMS) protocols [38]. A study by Amponsah and colleagues [24] found that training seminars were used by a district hospital in Ghana as an AMS program to train physicians to collect samples for culture and susceptibility analysis. In Nigeria, South Africa, Sudan and Ethiopia, laboratory staff have been trained in best practices of clinical specimen collection [42,51]. Moreover, to update pharmacists’ skills and knowledge of global changes and new trends in antibiotic prescribing, two hospitals in Ghana and Nigeria implemented a regular capacity building program for pharmacists as an AMR intervention [27,52]. Other health system inventions aim to raise awareness of AMR among the public and hospital staff [45,53].

#### 3.1.2 Infection prevention and control

These measures aim to limit disease transmission in healthcare settings, thereby reducing the need for antimicrobial intervention. The interventions carried out as part of infection prevention and control (IPC) include establishing hand hygiene protocols, maintaining appropriate use of personal protective equipment and implementing thorough cleaning and disinfection procedures. This intervention was the least mentioned among included studies. Amponsah and colleagues [24] noted that a university hospital in Ghana embedded infection prevention and control measures into AMS programing. As AMS team leader, the IPC specialist led the daily ward monitoring of antibiotic use guided by the hospital antibiotics guidelines and protocols [40].

#### 3.1.3 Monitoring and evaluation (guidelines, audit and feedback)

Regular monitoring of AMR patterns and trends plays a critical role in helping healthcare systems quickly identify emerging resistance and adapt their prescribing practices accordingly. The surveillance process includes various activities, such as tracking antibiotic dosages, reporting AMR data, adhering to AMS guidelines and protocols [30], and sharing best practices among healthcare facilities. This approach is widely practiced across African nations. The World Health Organization (WHO) advocates for the adoption of national guidelines to establish localized protocols for AMS programmes [54]. This method stands as the most potent tool, guiding physicians and pharmacists in making informed decisions concerning antibiotic prescriptions. As outlined by Sibande and colleagues [39], Queen Elizabeth Central Hospital (QECH) in Malawi created a tailored antibiotics guideline and subsequently uploaded it onto smartphones to provide guidance for clinicians in prescribing antibiotics. Similarly, Nayiga and colleagues [33] conducted a study to monitor the implementation of an AMS programme in a health centre in rural Uganda. Their findings indicated that the successful implementation of a locally tailored clinical guideline effectively assisted nurses in the appropriate dispensing of antibiotics to outpatient populations. In South Africa, hospitals have introduced the national AMS strategic framework and AMS hospital guidelines to oversee and ensure compliance with antibiotic prescriptions [40,41,44]. Hospitals in Kenya, Ghana, and Egypt have also implemented local hospital AMS guidelines [25,31,46]. To maintain rigorous adherence to these guidelines and protocols, routine perspective audits and feedback mechanisms [32,55] are consistently employed to assess antibiotic prescriptions. Additionally, protocols are periodically reviewed to identify outdated or obsolete guidelines [24,56].

#### 3.1.4 AMS team and support facilities

Beyond monitoring and evaluation, other health-system interventions identified in the included studies are the establishment of AMS teams and committees, the use of electronic devices, and the implementation of laboratory surveillance measures. Besset et al [23] investigated the use of electronic clinical decision making algorithms in a pediatric ward to ensure rational use of medicines, including antibiotics. The devices require the doctor to ask a series of questions about symptoms and vital clinical signs. Based on the information provided, possible diagnostic categories are then displayed. Similarly, El-Nile Badrawi Hospital in Egypt pioneered the creation of a mobile app device known as MEDIcare Pro. This innovative tool seamlessly integrates clinical and microbiological data gathered from routine diagnostic testing and analysis of laboratory findings [49]. To ensure effective implementation of the aforementioned AMS interventions, health centers in Ghana [36], Sierra Leone [53], Nigeria [22], Tanzania [36], Zambia [36], and Uganda[33] established AMS teams [35,36], a Drugs and Therapeutics Committee [57], and an AMS Subcommittee [53]. The interventions appear to be most effective primarily when there are accessible laboratory surveillance facilities in place. A cross-sectional survey conducted by Yopa et al. [28] uncovered that in Cameroon for example, sentinel laboratories play a crucial role in monitoring AMR profiles. They achieve this through conducting blood cultures to detect emerging resistant strains and subsequently reporting their findings to central laboratories. Additionally, a survey of hospitals in Nigeria revealed that 8 out of 25 hospitals reported performing blood cultures before dispensing antibiotics [21].

#### 3.1.5 Delay/Backup prescription

Another intervention identified during the review is delay/backup prescription. This method entails prescribing antibiotics to patients while also recommending a waiting period before patients obtain and use the medication. During this waiting period, the patient’s natural immune system is given an opportunity to combat the infection. If the patient’s condition deteriorates or fails to show improvement during this waiting period, they then proceed to obtain the prescription and commence antibiotic treatment. A study by Ghebrehewet et al. [26] in Ghana found that Ledzokuku Krowor Municipal Assembly (LEKMA) Hospital successfully implemented the delayed/backup prescribing model.

#### 3.1.6. Development of AMS action plan and team

The success of AMR interventions depend on the availability of AMS action plans and AMS teams to coordinate activities. Hall et al. [35] reported that Mbeya Zonal Referral Hospital in Tanzania successfully established an AMS team to develop local antimicrobial guidelines. Also, a five-year AMS action plan was developed in Sierra Leone to guide hospitals. The action plan included education, training, monitoring and surveillance [53].

### 3.2 Impact of AMR health-system interventions in Africa

The health-system interventions described have proven effective in reducing AMR. Four broad impacts of the described interventions were identified including 1. Reduction in antibiotics use, 2. Increased adherence to guidelines and protocols, 3. Enhanced laboratory-based AMR surveillance, 4. Development of AMS action plans and teams. The sub-sections below describe the impacts of some of the interventions in section 3.1.

#### 3.2.1 Reduction in antibiotics use

Several studies have provided evidence that AMR surveillance interventions, such as AMS programmes and educational campaigns advocating appropriate antibiotic utilisation, have the capacity to decrease antibiotic consumption. For example, El-Sokkary et al. [47] revealed that in Egypt the implementation of AMS programmes led to prudent use of antibiotics in hospitals. Findings from Sangeda et al. [32] revealed that AMR interventions in Tanzania resulted in the successful monitoring of antibiotic consumption. Similarly, in response to interventions, appropriate prescription of antibiotics in Ghana increased to 97.6% [25] and inappropriate use reduced by 8% [24]. In Tanzania, a training programme for over-the-counter drug sellers significantly increased good practice in dispensing antibiotics [37]. Additionally, the government of Zanzibar used financial incentives to influence good dispensing practices in health centres. This intervention increased antibiotics prescriptions made in adherence to the guidelines from 25.5% to 85.7% [30]. Moreover, Kerr et al. [38] reported that a training programme for pharmacists in Ghana increased antibiotics compliance from 18% to 70%. AMR interventions in Nigeria and Egypt reduced antibiotics consumption from 82.5% in 2015 to 51.1% in 2018 and 75.1 to 64.54 defined daily dose (DDD)/100 bed-days respectively. Also, defined daily doses of antibiotics in a South African hospital reduced from 1046 to 868 within two years [45]. Finally, regular audit and feedback systems are highly impactful in the reduction of antibiotics consumptions in Africa.

#### 3.2.2 Increased adherence to guidelines and protocols

Guidelines and antibiotics hospital protocols are critical to direct physicians’ prescriptionss in accordance with best practices. Bassiouny et al. [46] after monitoring compliance with antibiotics guidelines reported 86% adherence to new hospital antimicrobial guidelines. Sneddon et al. [52] reported that a capacity building programme for pharmacists in a Ghanaian hospital improved their behaviours and attitudes towards treatment guidelines. Finally, AMR interventions improved adherence to local and national AMS guidelines in Nigeria [55], and South Africa [41,44].

#### 3.2.3 Enhanced laboratory-based AMR surveillance

AMR surveillance interventions play a critical role in improving laboratory-based AMR surveillance and include activities such as antimicrobial susceptibility testing (AST) [47], laboratory data management and reporting. For example, a study conducted in Uganda showed that the implementation of a laboratory-based surveillance programme (LBS) resulted in noticeable improvements in antimicrobial AST, laboratory data management and reporting [34]. In Ghana, the number of blood sensitivity tests increased from 111 to 330 samples [24]. Furthermore, the availability of sentinel laboratories in Cameroon contributed to the early detection of AMR cases [28].

#### 3.2.4 Other impacts

Other impacts we have identified include awareness raising and behavioral and attitudinal changes among hospital staff. Education and training activities improve knowledge and awareness of AMS [50]. Lakoh et al. [53] reported that 10 pharmacist were successfully trained as champions for leading AMS activities. In Ghana, Sneddon et al. [52] observed changes in behaviours and attitudes of pharmacists after a training session. Another impact observed in Uganda and Zambia was that hospital administrators and managers joined the fight by leveraging on the training they received to produce alcohol-based hand sanitizers [38].

### 3.3 Challenges of implementing AMR health-system interventions

We identified two broad categories of challenges faced in implementing health system AMR interventions. In some instances, health systems lack the essential resources (financial, infrastructural, and human) required for the effective implementation of AMR interventions. This resulted in most interventions struggling to achieve their objectives. The broad challenges identified include; 1. Lack of resource, 2. Inadequate training and education and 3. Other impacts that do not neatly fall within the first two categories

#### 3.3.1 Lack of resources

One of the main challenges that has prevented health systems and AMS teams from successfully implementing interventions is the lack of essential resources such as finance, infrastructure (surveillance laboratories) and information Technology (IT) systems. Most of the AMR interventions lack financial support [40] to execute or organise training activities [34]. Additionally, infrequent meetings contribute to the obsolescence of AMR guidelines [33]. At lower-tier healthcare centres, the absence of surveillance laboratories for blood culture analysis [22,33] hampers informed diagnostic decision-making. Even when such facilities exist, they are often ill-equipped and inadequately structured to fully support AMR interventions [27], or lack IT systems for documentation and records keeping [32,34,51]. Financial limitations have also constrained health systems’ ability to acquire the necessary human resources to manage the increased workload associated with of AMS activities [31,37,43,56]. As a result, the available physicians are overburdened with AMS activities, undermining the effectiveness of AMR interventions.

#### 3.3.2 Inadequate training and education

Another challenge is that healthcare providers sometimes lack the necessary skills and knowledge to effectively implement ASP activities. Little or no training and education of key stakeholders in the healthcare system have resulted in poor awareness about AMR control strategies. A qualitative study in Nigeria revealed that many healthcare providers were not aware of AMR [57]. Yopa et al. [28] noted that lack of regular training of staff resulted in poor data entry and transmission at laboratories in Cameroon. In Malawi, physicians and medical interns made inaccurate diagnoses due to inadequate knowledge of local antibiotic guidelines. Furthermore, leadership support for AMR interventions has been very low due to poor education on AMR activities in South Africa [40,45], Nigeria [57] and Ghana [26].

#### 3.3.3 Other challenges

We identified other challenges that hampered the implementation of AMR interventions. A Nigerian study showed low blood culture utilisation. [21]. This challenge posed a difficulty in prescribing the appropriate antibiotics. Another challenge was that COVID-19 disrupted the implementation of some AMR interventions. For example, reductions in antibiotic consumption in Ghana could not be sustained due to COVID restrictions [24]. In addition, the restrictions also limited AMS team members’ meeting times [29,32,44].

## 4. Discussion

We conducted a scoping review to identify health system AMR interventions, their impacts and the challenges faced in implementation processes in Africa. The fifteen African health systems where studies were conducted are contextually varied with several key differences including in health workforce strength and other existing infrastructure for implementing AMR interventions. For example, whereas in almost all the countries under study National Action Plans (NAP) for AMR have been integrated with other sectors, in countries such as Malawi and Uganda this is not yet the case. Additionally, recent data on whether countries had established or were starting to establish surveillance systems for AMR in human health revealed that countries are in very different stages of this process. Whereas countries like Malawi, Tanzania, Ghana, Zambia, Tunisia, Cameroon, Burkina Faso, Kenya and Ethiopia have established surveillance systems, countries such as South Africa, Uganda, Nigeria, Egypt, Sudan and Sierra Leone have not. Interestingly all the countries included in this study except for Cameroon offer some form of training for health workers on AMR although in most cases this training is rather Ad Hoc. These differences offer context for understanding the health system interventions described.

The findings of the review indicate that education, training and capacity building are mostly implemented as AMS programmes in Africa. Education and awareness creation is crucial for both health care providers and patients [58,59]. According to WHO, outpatients with good information on AMR are likely to avoid the abuse and misuse of antibiotics [60]. In contrast, this review revealed that education and training initiatives primarily targeted pharmacists, physicians, and laboratory technicians, with less attention to patients. However, it is important to underscore that despite their limitations, these educational and training endeavours have had a positive impact, equipping healthcare providers with the skills necessary to effectively lead and execute successful AMS programmes [61]. It is worth emphasizing that the absence of education and training played a pivotal role in the shortcomings of health system interventions in Africa [62]. Audit and feedback were also identified as an impactful AMR intervention. According to Vanstone et al [63] audit and feedback as monitoring interventions improved the prescription of antibiotics by primary healthcare prescribers. Glenngård and Anell [64] also noted that audit and feedback improves the quality of healthcare delivery.

One of the primary objectives of this review was to assess the impact of the health system interventions in combating AMR in Africa. A significant discovery from this review was the observed counterintuitive increase in antibiotic consumption among outpatients in Uganda following the implementation of AMS guidelines. One of the key objectives of health system AMR interventions is to regulate and enhance antibiotic prescriptions. Unfortunately, this specific intervention fell short of achieving its intended goal due to the unavailability of blood culture laboratories to guide prescription decisions. Singular and siloed interventions may not be sufficient to curb the widespread proliferation of AMR. Health facilities that successfully implemented multidimensional AMR interventions were able to significantly reduce antibiotic consumption. Based on evidence from Asia, Holloway et al. [65] have reported on the importance of use of multiple interventions such as hospital drug and therapeutic committees and public education on antibiotic use. Moreover, a study conducted in four private tertiary hospitals in Saudi Arabia revealed that the introduction of AMS initiatives resulted in a significant decline in the utilisation of antimicrobials. Additionally, the implementation of these programmes was found to be correlated with a decrease in the occurrence of healthcare-associated infections (HAIs). This study highlights the effectiveness of AMS programmes in mitigating the rise of AMR and HAIs, leading to financial benefits associated with the prudent utilisation of antimicrobials [66].

In the context of AMS programmes, Information Technology (IT) plays a crucial role in facilitating the creation of electronic surveillance systems. These systems are designed to monitor both the utilisation of antimicrobials and the patterns of resistance at both national and regional levels [67]. The results of this review unveiled a critical issue wherein surveillance laboratories were unable to track emerging patterns of AMR due to inadequate IT infrastructure. Furthermore, the review established that AMR interventions faltered because healthcare facilities lacked the necessary IT systems for maintaining records of patient data, treatment histories, and prescription details. The absence of such information systems pose a significant hindrance to AMS teams in their efforts to monitor AMR surveillance effectively [68].

Despite the recognized value and significance of health system interventions in mitigating the global spread of AMR, their implementation encountered notable challenges that require attention from healthcare system stakeholders. The findings highlight a significant hurdle: the inadequacy of resources for AMS programmes. AMS teams grapple with inadequate financial support and logistical resources, impeding their ability to effectively execute these programmes. A study conducted in India revealed several obstacles associated with the implementation of sustainable stewardship programmes. This includes competition among doctors, time constraints faced by physicians, absence of AMS champions, sub-optimal interdepartmental cooperation, absence of supporting facilities, dysfunctional regulatory systems, and unreliability of antibiograms [69]. In low-and middle-income countries (LMICs), AMS programmes encounter many problems. These challenges include insufficient funding, inadequate access to suitable technologies, overburdened healthcare systems, poor knowledge and awareness, and a scarcity of qualified personnel.

It is important to acknowledge certain limitations of this review. Notably, the review excluded articles not published in English, which may have inadvertently omitted research from some Francophone, Lusophone, Hispanophone and Arabophone African countries. There remains several gaps in knowledge on how and under what circumstances the described health system interventions are effective. For example, are health worker training and education programs effective in contexts that lack laboratory infrastructure or information technology? Additionally, does the effectiveness of health system related AMR interventions depend on widespread health worker and public awareness of AMR? Such questions might be explored through realist reviews to reveal how contexts and mechanisms influence the effectiveness of the health system interventions described.

## 5. Conclusion

Health system interventions have demonstrated some effectiveness in containing AMR. Nevertheless, these interventions face challenges, including resource and logistical limitations, inadequate education and training, and limited leadership commitment. The results of this study can serve as a valuable resource for policy makers by highlighting the importance of AMR interventions and barriers to implementation. This awareness can, in turn, promote more effective implementation of interventions across Africa.

## Supporting information

S1 Checklist PRISMA-ScR checklist. (DOCX)
